# Urban vs. rural: colorectal cancer survival and prognostic disparities from 2000 to 2019

**DOI:** 10.3389/fpubh.2024.1319977

**Published:** 2024-02-09

**Authors:** Ming-sheng Fu, Shu-xian Pan, Xun-quan Cai, Qin-cong Pan

**Affiliations:** ^1^Department of Gastroenterology, Shanghai Fifth People's Hospital Fudan University, Shanghai, China; ^2^Department of Anesthesiology, Shanghai Fifth People's Hospital Fudan University, Shanghai, China

**Keywords:** colorectal cancer, survival, prognostic, urban-rural, surveillance, epidemiology, end results

## Abstract

This study aimed to analyze the differences in colorectal cancer (CRC) survival between urban and rural areas over the past 20 years, as well as investigate potential prognostic factors for CRC survival in both populations. Using registry data from Surveillance, Epidemiology, and End Results (SEER) from 2000 to 2019, 463,827 CRC cases were identified, with 85.8% in urban and 14.2% in rural areas. The mortality of CRC surpassed its survival rate by the sixth year after diagnosis in urban areas and the fifth year in rural areas. Furthermore, the 5-year overall survival (OS) of CRC increased by 2.9–4.3 percentage points in urban and 0.6–1.5 percentage points in rural areas over the past two decades. Multivariable Cox regression models identified independent prognostic factors for OS and disease-specific survival (DSS) of CRC in urban and rural areas, including age over 40, Black ethnicity, and tumor size greater than 5 cm. In addition, household income below $75,000 was found to be an independent prognostic factor for OS and DSS of CRC in urban areas, while income below $55,000 was a significant factor for rural areas. In conclusion, this study found a notable difference in CRC survival between rural and urban areas. Independent prognostic factors shared among both rural and urban areas include age, tumor size, and race, while household income seem to be area-specific predictive variables. Collaboration between healthcare providers, patients, and communities to improve awareness and early detection of CRC may help to further advance survival rates.

## Introduction

Colorectal cancer (CRC) is the third most common cancer worldwide, the incidence of CRC in China is rising continuously in recent years (1). In the United States, CRC is the third most commonly diagnosed cancer and the third most common cause of cancer-related death. In 2023, It has been estimated that 106,970 cases of colon cancer and 46,050 cases of rectal cancer will be newly diagnosed in the US, and a total of 52,550 people will die from these cancers (2). We know that the risk factors that can change CRC mortality include smoking, an unhealthy diet, high alcohol consumption, lack of exercise, and overweight. In addition, regular screening, monitoring and high-quality treatment can reduce the incidence rate and mortality of CRC (3). Although the prognosis of colorectal cancer has improved over the years due to advances in diagnosis and treatment options, the mortality rate of colorectal cancer has decreased significantly since 1975 (4). However, to date, no study has compared CRC survival and prognosis trends between urban and rural areas over the past two decades, the differences in CRC survival between urban and rural areas over the past 20 years is unclear, and the potential prognostic factors for CRC survival in urban and rural areas is unclear. To address this gap, by evaluating CRC survival data, we aimed to investigate differences in survival and prognosis between the urban and rural populations from 2000 to 2019, and investigate potential prognostic factors for CRC survival in both populations.

The objective of this study is to present an analysis of the prognostic patterns of CRC in both urban and rural regions over the past two decades, as well as exploring possible factors that could impact CRC survival rates in each location. Such findings could be vital in highlighting divergences existing in screening and treatment methods for CRC patients in urban and rural areas, ultimately helping in creating equitable access to quality cancer care, regardless of where a patient resides.

## Materials and methods

### Data source

The Surveillance, Epidemiology, and End Results (SEER) database, established by the National Cancer Institute (NCI), was utilized to gather patient records encompassing clinicopathological information such as occurrence, treatment, and survival data for various tumors. For this study, the SEER*Stat software (version 8.4.0.1) was implemented to obtain data from the “Incidence-SEER Research Data, 17 Registries, Nov 2021 Sub (2000–2019)” database.

### Patients

Patients who were diagnosed with CRC from 2000 to 2019 were screened out from the database. Patients whom we selected met the following conditions: {Site and Morphology. Site recode ICD-O-3/WHO 2008} = “Colon and Rectum”, and {Race, Sex, Year Dx. Year of diagnosis} = “2000–2019”, and {Site and Morphology.ICD-O-3 Hist/behav} = “8140/3: Adenocarcinoma, NOS”, and {Site and Morphology. Diagnostic Confirmation} = “Positive histology”, and excluded race recode “Unknown” cases and Survival months “Unknown” cases, Finally, 463,827 colorectal adenocarcinoma cases were included in the study.

### Study variables

Clinical variables including age (<40 years; 40-64 years; ≧65 years), sex (male and female), race (White W; Black B; American Indian/Alaska Native AI; Asian or Pacific Islander API), year of diagnosis (2000–2019), primary site [rectum includes rectum and rectum colon junction (RRSJ); left colon includes sigmoid colon, descending colon and splenic flexure of colon (SDS); right colon includes transverse colon, ascending colon, hepatic flexure of colon (TAH) and cecum, Appendix (CA)], stage (0, I; II; III; IV; Unknown), tumor size (<5 cm and ≧5 cm), median household income (>$75,000; $55,000–$75,000; $35,000–$55,000; <$35,000), rural (Adjacent to a metropolitan; Not adjacent to a metropolitan)-urban (1 million pop, 250,000 to 1 million pop, 250 thousand pop), Status (Alive and Dead), Cause-specific death (Dead of this cancer; Dead of other cause) were used in the current study. AJCC stage 3rd edition (1988–2003) is applicable to the stage of diagnosing CRC in 2000–2003, Derived AJCC Stage Group, 6th ed. (2004–2015) is applicable to the stage of diagnosing CRC in 2004–2015, Derived SEER Cmb Stg Grp (2016–2017) is applicable to the stage of diagnosing CRC in 2016–2017, and Derived EOD 2018 Stage Group (2018+) is applicable to the stage of diagnosing CRC in 2018–2019. EOD 10-size (1988–2003) is applicable to the Tumor size of CRC diagnosed in 2000–2003, CS Tumor size (2004–2015) is applicable to the Tumor size of CRC diagnosed in 2004–2015, and Tumor Size Summary (2016+) is applicable to the Tumor size of CRC diagnosed in 2016-2019. Both overall survival (OS) and disease-specific survival (DSS) were used to analyze the survival outcomes.

### Statistical analysis

The study used descriptive statistics to summarize demographic information and performed a chi-square test to compare categorical variables between urban and rural cases as baseline clinical characteristics. The SEER cause-specific death classification was utilized to determine the time at which patients who died from cancer were censored for DSS analyses, while patients who died from any cause were also censored for OS analyses. Using Kaplan–Meier for survival analysis. GraphPad Prism 8 (GraphPad Software, La Jolla, CA, United States) survival curves were employed to analyze both OS and DSS, and these were compared using the Log-rank (Mantel-Cox) test. Moreover, the study utilized univariate and multivariable Cox proportional hazards regression models to analyze the prognostic factors of OS and DSS for CRC.

The SEER Stat (National Cancer Institute, Bethesda, MD, United States; version 8.4.0.1) was used to download data in this study. Statistical analyses were performed using IBM SPSS Statistics version 25 (IBM Corporation, Armonk, NY, United States). All analyses were double-sided, and a *p* value < 0.05 was deemed statistically significant.

## Results

### Distribution of CRC in urban and rural areas

The study analyzed a total of 463,827 cases of colorectal cancer (CRC) between 2000 and 2019, with 85.8% being reported in urban areas and 14.2% in rural areas. In the past two decades, the proportion of CRC diagnosed before the age of 40 was slightly higher 0.7 percentage points in urban areas than in rural areas. Conversely, the proportion of CRC aged 65 or older was found to be 1.4 percentage points higher in rural areas than in urban areas. The proportion of women with CRC was higher 1.4 percentage points in urban areas compared to rural areas. When examining racial demographics, Black and Asian or Pacific Islander populations were, respectively, found to be higher 2.6 and 6.0 percentage points in urban areas as compared to rural areas. On the other hand, White and American Indian populations were found to be higher 7.5 and 2.1 percentage points in rural areas respectively, compared to urban areas.

Regarding clinical findings, the proportion of CRC cases diagnosed at stage III and stage IV was higher 1.6 percentage points in urban areas than in rural areas. Additionally, the proportion of tumors less than 5 cm in size was higher 0.8 percentage points in urban areas compared to rural areas. In terms of household income, 85% of households with incomes over $55,000 were located in urban areas compared to only 24.2% in rural areas. Finally, the total mortality rate of CRC was found to be higher 3.5 percentage points in rural areas compared to urban areas, while CRC-specific mortality rates were higher 1.9 percentage points in rural areas compared to urban areas (as shown in Table 1).

**Table 1:** Comparison between urban and rural areas.

Variable		Urban		Rural		*X* ^2^	*p*-value
		*N* (%)		*N* (%)			
Age	5–39 years	10,231	2.6%	1,234	1.9%	139.18	<0.0001
	40–64 years	146,274	36.8%	23,716	36.0%		
	≧65 years	241,458	60.7%	40,914	62.1%		
Sex	Female	189,501	47.6%	30,404	46.2%	48.05	<0.0001
	Male	208,462	52.4%	35,460	53.8%		
Race	White	314,470	79.0%	57,003	86.5%	7258.95	<0.0001
	Black	44,634	11.2%	5,685	8.6%		
	AI	1,765	0.4%	1,635	2.5%		
	API	37,094	9.3%	1,541	2.3%		
Primary site	RRSJ	116,198	29.2%	19,180	29.1%	9.59	0.022
	SDS	121,001	30.4%	19,760	30.0%		
	TAH	97,943	24.6%	16,249	24.7%		
	CA	62,821	15.8%	10,675	16.2%		
Stage	0	4,363	1.1%	793	1.2%	76.26	<0.0001
	I	66,039	16.6%	11,370	17.3%		
	II	109,285	27.5%	18,204	27.6%		
	III	109,385	27.5%	17,469	26.5%		
	IV	83,142	20.9%	13,397	20.3%		
	Unknown	25,749	6.5%	4,631	7.0%		
Tumor size	<5 cm	189,160	47.5%	30,740	46.7%	16.77	<0.0001
	≧5 cm	208,803	52.5%	35,124	53.3%		
Median household income	>$75,000	140,550	35.3%	2,937	4.5%	138121.15	<0.0001
	$55,000–$75,000	200,471	50.4%	12,996	19.7%		
	$35,000–$55,000	56,522	14.2%	42,329	64.3%		
	<$35,000	420	0.1%	7,602	11.5%		
Status	Dead	229,987	57.8%	40,384	61.3%	288.52	<0.0001
	Alive	167,976	42.2%	25,480	38.7%		
Cause-specific death	Dead of this cancer	143,244	36.0%	24,938	37.9%	292.82	<0.0001
	Dead of other cause	86,743	21.8%	15,446	23.5%		
	Alive	167,976	42.2%	25,480	38.7%		

AI, American Indian/Alaska Native; API, Asian or Pacific Islander; RRSJ, Rectum, Rectosigmoid junction; SDS, Sigmoid, Descending, Splenic flexure of colon; TAH, Transverse, Ascending, Hepatic flexure of colon; CA, Cecum, Appendix.

### Kaplan–Meier survival analysis

The Kaplan Meier survival analysis revealed that the median survival months for CRC were higher in urban areas compared to rural areas. The median survival of age groups of less than 40, 40–64 and over 65 in urban areas had a higher 8.7 months, 4.4 months, and 2.3 months respectively, when compared to their rural counterparts. When analyzing the effect of household income on CRC’s prognosis, The median survival of household earning more than $75,000 *per annum* had a higher 4 months in urban compared rural. Conversely, the median survival of those earning less than $35,000 annually had a lower 9 months in urban when compared to their rural counterparts (as shown in Table 2).

**Table 2:** Kaplan–Meier survival analysis.

Variable		Urban	Rural	*X* ^2^	*p*-value
		Median survival months (95% CI)	Median survival months (95% CI)		
Age	5–39 years	142.64 (140.31–144.97)	138.24 (131.69–144.80)	28398.51	<0.0001
	40–64 years	131.10 (130.50–131.70)	122.40 (120.97–123.84)		
	≧65 years	74.87 (74.52–75.23)	72.56 (71.73–73.39)		
Sex	Female	97.50 (97.03–97.98)	93.75 (92.60–94.90)	92.28	<0.0001
	Male	94.42 (93.97–94.87)	88.49 (87.45–89.53)		
Race	White	95.25 (94.89–95.61)	91.47 (90.64–92.29)	1299.20	<0.0001
	Black	87.83 (86.85–88.81)	82.81 (80.19–85.430)		
	AI	101.27 (95.93–106.61)	96.39 (91.09–101.70)		
	API	112.46 (111.28–11,364)	96.25 (91.19–101.31)		
Primary site	RRSJ	101.25 (100.63–101.88)	94.96 (93.51–96.42)	909.28	<0.0001
	SDS	97.22 (96.62–97.82)	91.18 (89.77–92.59)		
	TAH	93.14 (92.50–93.79)	89.48 (87.95–91.00)		
	CA	87.96 (87.17–88.74)	85.37 (83.52–87.22)		
Stage	0	123.94 (120.48–127.40)	113.07 (105.34–120.80)	134540.85	<0.0001
	I	132.99 (132.18–133.82)	124.42 (122.50–126.34)		
	II	118.04 (117.42–118.65)	113.06 (111.61–114.51)		
	III	109.08 (108.43–109.72)	102.23 (100.69–103.77)		
	IV	28.75 (28.36–29.15)	26.26 (25.35–27.16)		
	Unknown	60.12 (59.01–61.23)	62.20 (59.67–63.74)		
Tumor size	<5 cm	110.74 (110.26–111.22)	104.99 (103.85–106.13)	11972.86	<0.0001
	≧5 cm	82.34 (81.90–8,278)	78.49 (77.47–79.52)		
Median household income	>$75,000	99.96 (99.40–100.51)	95.98 (92.32–99.63)	418.96	<0.0001
	$55,000–$75,000	94.14 (93.68–94.59)	93.68 (91.97–95.39)		
	$35,000–$55,000	91.87 (90.98–92.76)	90.23 (89.27–91.18)		
	<$35,000	79.16 (71.53–86.80)	88.17 (85.72–90.60)		

### Comparison of the OS and DSS of CRC between urban and rural areas in 20 years

**Figure 1:**
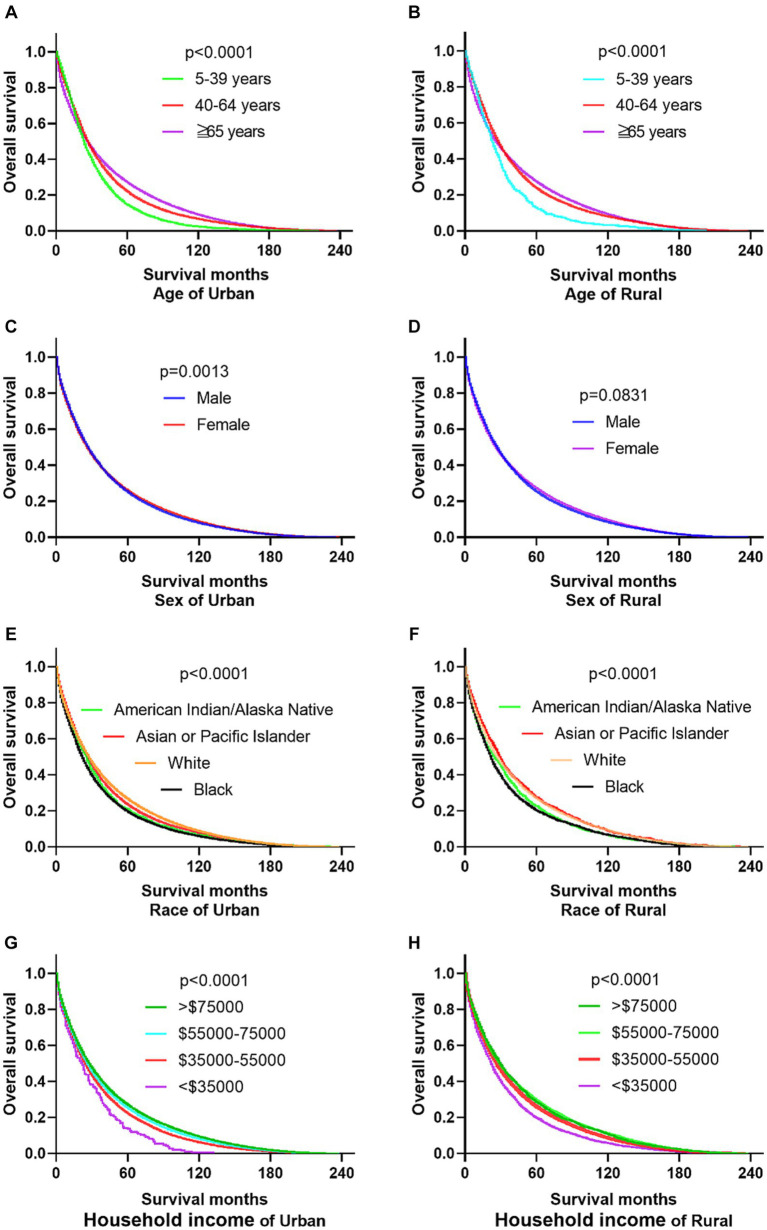
Comparative OS of CRC: age, sex, race, and household income factors in urban and rural areas. **(A,B)** In urban and rural areas, CRC patients under the age of 40 have the worst OS. **(C,D)** The OS of male CRC patients in urban areas is worse than that of female patients, while there is no significant difference OS between male and female patients in rural areas. **(E,F)** Black CRC patients have the lowest OS in urban and rural areas. **(G,H)** CRC patients with incomes exceeding $75,000 in urban and rural households have the highest OS, while CRC patients with incomes below $35,000 have the lowest OS.

**Figure 2:**
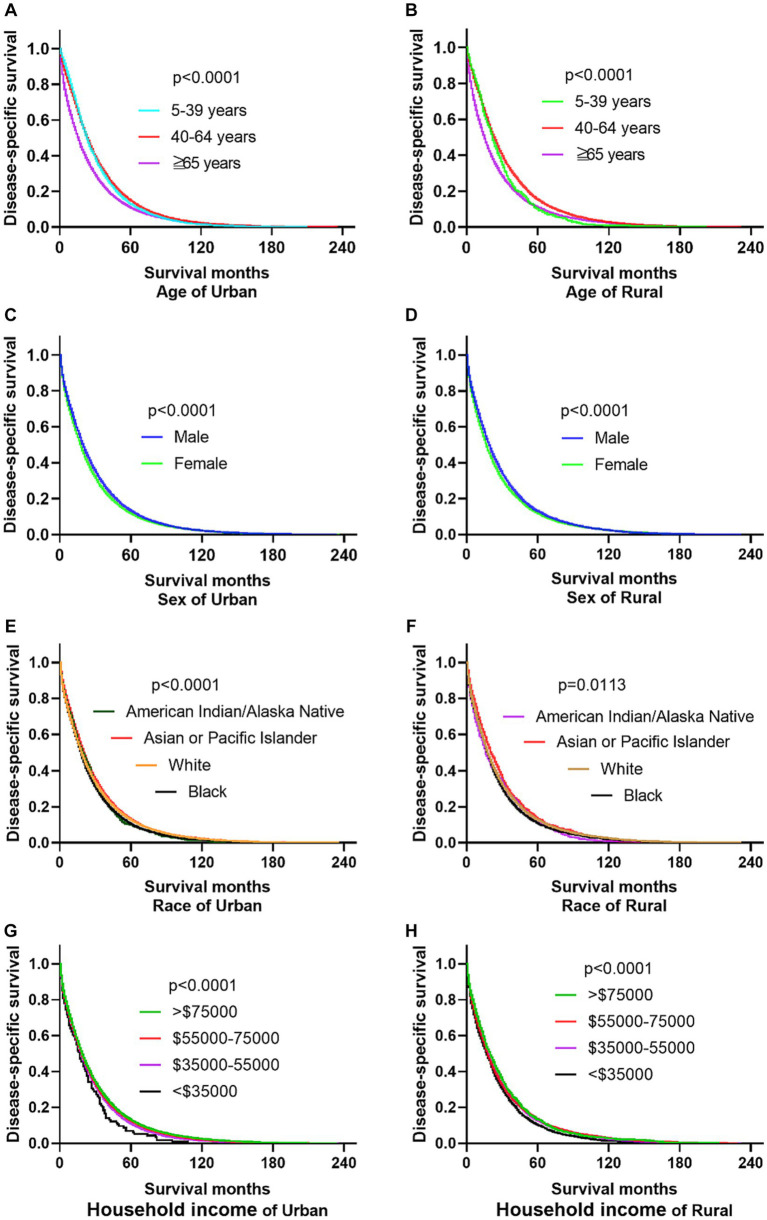
Comparative DSS of CRC: age, gender, ethnicity, and income factors in urban and rural areas. **(A,B)** Urban and rural CRC patients over 65 years old have the worst DSS. **(C,D)** The DSS of CRC women in urban and rural areas is lower than that of men. **(E,F)** The DSS of black people in urban and rural areas is the lowest. **(G,H)** CRC patients with households incomes below $35,000 in urban and rural have the lowest DSS.

The research results show that in urban and rural areas, CRC patients under 40 years old have the worst OS, while CRC patients over 65 years old have the worst DSS. The OS of male CRC patients in urban areas is worse than that of female patients, while there is no significant difference in OS between male and female patients in rural areas. Whether in urban or rural areas, the DSS of female CRC patients is slightly lower than that of male patients, while the OS and DSS of black and family income below $35,000 CRC patients are the lowest (as shown in Figures 1, 2).

**Figure 3:**
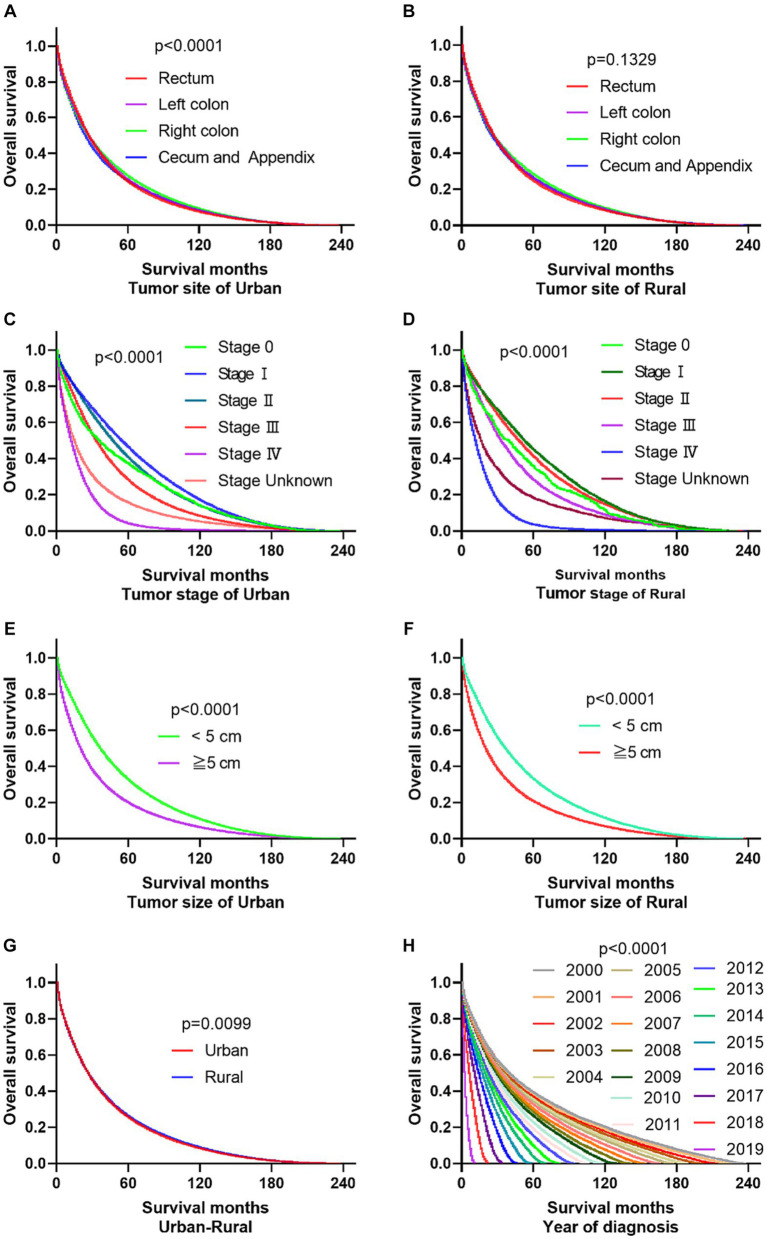
Comparative OS of CRC: primary site, stage, tumor size, urban–rural and year of diagnosis. **(A,B)** The OS of CRC patients with primary site Rectum and Cecum and Appendix was the worst in urban, it is not significantly different compared the primary site in rural. **(C,D)** Whether in urban or rural, the OS of CRC patients in Stage IV and Stage Unknown were significantly reduced. **(E,F)** No matter in urban or rural, the OS of CRC patients with tumors over 5 cm was significantly reduced. **(G)** The OS of urban CRC patients is slightly lower than that of rural patients. **(H)** Comparison of OS in CRC patients diagnosed in urban and rural areas from 2000 to 2019.

**Figure 4:**
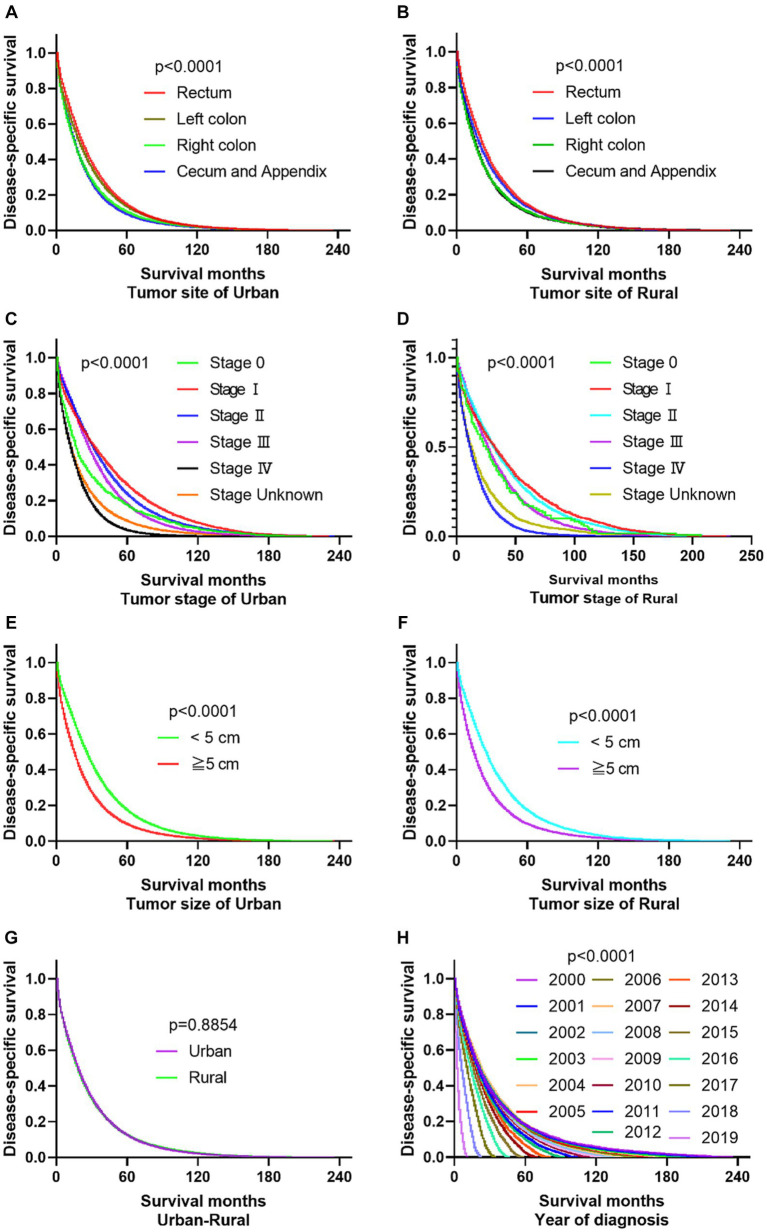
Comparative DSS of CRC: Primary Site, Stage, Tumor Size, Urban–Rural and year of diagnosis. **(A,B)** The DSS of CRC patients with primary site Right colon and Cecum and Appendix was the worst in urban and rural. **(C,D)** No matter in urban or rural, the DSS of CRC patients in Stage IV and Stage Unknown were significantly reduced. **(E,F)** The DSS of CRC patients with tumors over 5 cm was significantly reduced. **(G)** There was no significant difference in DSS between urban and rural CRC patients. **(H)** Comparison of DSS in CRC patients diagnosed in urban and rural areas from 2000 to 2019.

The primary site of cancer also played a significant role in survival outcomes. For instance, CRC patients diagnosed with primary site Rectum and Cecum and Appendix in urban areas had the worst OS, those with primary site Right colon and Cecum and Appendix had the worst DSS. Patients identified as Stage IV and Stage Unknown had significantly reduced OS and DSS. Interestingly, patients with tumors larger than 5 cm demonstrated significantly reduced OS and DSS rates in both urban and rural settings. Notably, the OS of urban CRC patients is slightly lower than that of rural patients, while, there was no significant difference in DSS between urban and rural CRC patients (as shown in Figures 3, 4).

### Independent prognostic factors for OS and DSS of CRC

This study conducted a Cox proportional hazard model to analyze the risk factors associated with the survival of CRC patients. Univariate analysis showed that several variables significantly impacted the risk of death for CRC patients. These included age over 65 years old, male gender, Black race, tumor location (ileocecal tumors had the worst prognosis compared to rectum), tumor stage (stage III, IV, and unknown), and tumor size (tumors over 5 cm had a higher risk of death). In addition, the study found that household income also had a significant impact on CRC survival, with those earning less than $55,000 having a decreased survival rate. The rural–urban divide was also examined, and it was found that the survival of CRC in rural areas was slightly lower than that in urban areas (as shown in Tables 3, 4).

**Table 3:** Univariate analysis of overall survival using Cox proportional hazards models.

Variable		*N*	HR	95% CI	*p*-value
Age	5–39 years	11,465	REF		
	40–64 years	169,990	1.152	1.117–1.189	<0.0001
	≧65 years	282,372	2.305	2.236–2.376	<0.0001
Sex	Female	219,905	REF		
	Male	243,922	1.038	1.030–1.046	<0.0001
Race	White	371,473	REF		
	Black	50,319	1.127	1.127–1.141	<0.0001
	AI	3,400	0.957	0.914–1.002	0.059
	API	38,635	0.807	0.807–0.819	<0.0001
Primary site	RRSJ	135,378	REF		
	SDS	140,761	1.068	1.058–1.079	<0.0001
	TAH	114,192	1.107	1.096–1.119	<0.0001
	CA	73,496	1.186	1.173–1.200	<0.0001
Stage	0	5,156	REF		
	I	77,409	0.855	0.818–0.893	<0.0001
	II	127,489	1.045	1.001–1.091	0.046
	III	126,854	1.209	1.158–1.263	<0.0001
	IV	96,539	4.708	4.510–4.915	<0.0001
	Unknown	30,380	2.544	2.433–2.660	<0.0001
Tumor size	<5 cm	219,900	REF		
	≧5 cm	243,927	1.523	1.511–1.534	<0.0001
Median household income	>$75,000	143,487	REF		
	$55,000–$75,000	213,467	1.081	1.071–1.090	<0.0001
	$35,000–$55,000	98,851	1.121	1.109–1.133	<0.0001
	<$35,000	8,022	1.169	1.135–1.205	<0.0001
Rural–Urban areas	Urban	397,963	REF		
	Rural	65,864	1.064	1.053–1.075	<0.0001

AI, American Indian/Alaska Native; API, Asian or Pacific Islander; RRSJ, Rectum, Rectosigmoid junction; SDS, Sigmoid, Descending, Splenic flexure of colon; TAH, Transverse, Ascending, Hepatic flexure of colon; CA, Cecum, Appendix; HR, hazard ratio; CI, confidence interval.

**Table 4:** Univariate analysis of disease-specific survival using Cox proportional hazards models.

Variable		*N*	HR	95% CI	*p*-value
Age	5–39 years	11,465	REF		
	40–64 years	169,990	1.002	0.970–1.036	0.886
	≧65 years	282,372	1.323	1.281–1.366	<0.0001
Sex	Female	219,905	REF		
	Male	243,922	1.031	1.022–1.041	<0.0001
Race	White	371,473	REF		
	Black	50,319	1.273	1.254–1.291	<0.0001
	AI	3,400	1.043	0.986–1.103	0.142
	API	38,635	0.891	0.875–0.907	<0.0001
Primary site	RRSJ	135,378	REF		
	SDS	140,761	1.018	1.006–1.031	0.003
	TAH	114,192	0.886	0.874–0.898	<0.0001
	CA	73,496	1.038	1.023–1.053	<0.0001
Stage	0	5,156	REF		
	I	77,409	0.67	0.624–0.719	<0.0001
	II	127,489	1.114	1.039–1.194	0.002
	III	126,854	1.958	1.827–2.098	<0.0001
	IV	96,539	9.568	8.930–10.251	<0.0001
	Unknown	30,380	4.056	3.780–4.352	<0.0001
Tumor size	<5 cm	219,900	REF		
	≧5 cm	243,927	1.895	1.876–1.914	<0.0001
Median household income	>$75,000	143,487	REF		
	$55,000–$75,000	213,467	1.086	1.074–1.099	<0.0001
	$35,000–$55,000	98,851	1.124	1.109–1.139	<0.0001
	<$35,000	8,022	1.179	1.136–1.224	<0.0001
Rural–Urban areas	Urban	397,963	REF		
	Rural	65,864	1.056	1.042–1.070	<0.0001

Upon conducting multivariate analysis, it was found that age over 40 years, male gender, Black race, right colon tumor location, stage III or IV, and tumors over 5 cm were independent prognostic factors for OS in both urban and rural settings. Age over 40 years, Black race, and tumors over 5 cm were identified as independent prognostic factors for DSS. Household income also played a role, as income less than $75,000 and less than $55,000 were independent prognostic factors for OS and DSS of CRC in urban and rural areas, respectively (as shown in Tables 5, 6).

**Table 5:** Multivariate analysis comparing the risk factors of overall survival in urban and rural.

Variable		Urban			Rural		
		HR	59% CI	*p*-value	HR	59% CI	*p*-value
Age	5–39 years	REF			REF		
	40–64 years	1.269	1.228–1.312	<0.0001	1.281	1.17–1.402	<0.0001
	≧65 years	3.046	2.948–3.147	<0.0001	2.831	2.589–3.095	<0.0001
Sex	Female	REF			REF		
	Male	1.056	1.048–1.065	<0.0001	1.076	1.055–1.097	<0.0001
Race	White	REF			REF		
	Black	1.123	1.108–1.138	<0.0001	1.138	1.099–1.178	<0.0001
	AI	0.970	0.909–1.035	0.356	1.114	1.036–1.197	0.003
	API	0.852	0.839–0.865	<0.0001	0.992	0.927–1.061	0.809
Primary site	RRSJ	REF			REF		
	SDS	1.008	0.997–1.019	0.147	1.026	1.0–1.053	0.047
	TAH	1.072	1.06–1.085	<0.0001	1.073	1.044–1.103	<0.0001
	CA	1.078	1.064–1.092	<0.0001	1.065	1.033–1,099	<0.0001
Stage	0	REF			REF		
	I	0.887	0.846–0.931	<0.0001	0.883	0.793–0.983	0.023
	II	1.038	0.99–1.088	0.126	0.980	0.881–1.089	0.706
	III	1.341	1.279–1.405	<0.0001	1.262	1.135–1.403	<0.0001
	IV	5.469	5.217–5.734	<0.0001	4.971	4.471–5.526	<0.0001
	Unknown	2.511	2.392–2.637	<0.0001	2.081	1.866–2.322	<0.0001
Tumor size	<5 cm	REF			REF		
	≧5 cm	1.262	1.252–1.273	<0.0001	1.272	1.246–1.298	<0.0001
Median household income	>$75,000	REF			REF		
	$55,000–$75,000	1.073	1.063–1.083	<0.0001	1.011	0.956–1.069	0.705
	$35,000–$55,000	1.098	1.084–1.113	<0.0001	1.072	1.017–1.131	0.01
	<$35,000	1.003	0.876–1.15	0.96	1.138	1.072–1.208	<0.0001

AI, American Indian/Alaska Native; API, Asian or Pacific Islander; RRSJ, Rectum, Rectosigmoid junction; SDS, Sigmoid, Descending, Splenic flexure of colon; TAH, Transverse, Ascending, Hepatic flexure of colon; CA, Cecum, Appendix; HR, hazard ratio; CI, confidence interval.

**Table 6:** Multivariate analysis comparing the risk factors of disease-specific survival in urban and rural.

Variable		Urban			Rural		
		HR	59% CI	*p*-value	HR	59% CI	*p*-value
Age	5–39 years	REF			REF		
	40–64 years	1.170	1.13–1.211	<0.0001	1.136	1.033–1.25	0.008
	≧65 years	2.058	1.988–2.13	<0.0001	1.868	1.7–2.053	<0.0001
Sex	Female	REF			REF		
	Male	1.002	0.991–1.012	0.718	1.012	0.986–1.037	0.371
Race	White	REF			REF		
	Black	1.179	1.161–1.198	<0.0001	1.159	1.111–1.209	<0.0001
	AI	0.999	0.924–1.08	0.982	1.126	1.031–1.231	0.009
	API	0.907	0.89–0.924	<0.0001	1.015	0.933–1.104	0.73
Primary site	RRSJ	REF			REF		
	SDS	0.965	0.953–0.978	<0.0001	0.988	0.957–1.02	0.445
	TAH	0.970	0.956–0.985	<0.0001	0.978	0.944–1.013	0.215
	CA	1.024	1.008–1.041	0.004	1.004	0.966–1.044	0.831
Stage	0	REF			REF		
	I	0.702	0.65–0.759	<0.0001	0.817	0.683–0.978	0.028
	II	1.113	1.032–1.201	0.005	1.141	0.957–1.361	0.141
	III	2.109	1.956–2.274	<0.0001	2.192	1.84–2.611	<0.0001
	IV	10.325	9.578–11.13	<0.0001	10.281	8.634–12.242	<0.0001
	Unknown	3.817	3.535–4.122	<0.0001	3.546	2.967–4.238	<0.0001
Tumor size	<5 cm	REF			REF		
	≧5 cm	1.376	1.361–1.391	<0.0001	1.385	1.349–1.423	<0.0001
Median household income	>$75,000	REF			REF		
	$55,000–$75,000	1.082	1.07–1.095	<0.0001	1.073	0.998–1.154	0.056
	$35,000–$55,000	1.092	1.074–1.11	<0.0001	1.135	1.059–1.216	<0.0001
	<$35,000	0.872	0.736–1.034	0.116	1.178	1.091–1.272	<0.0001

### Trends changes of CRC cases, survival and mortality in urban and rural areas in 20 years

**Figure 5:**
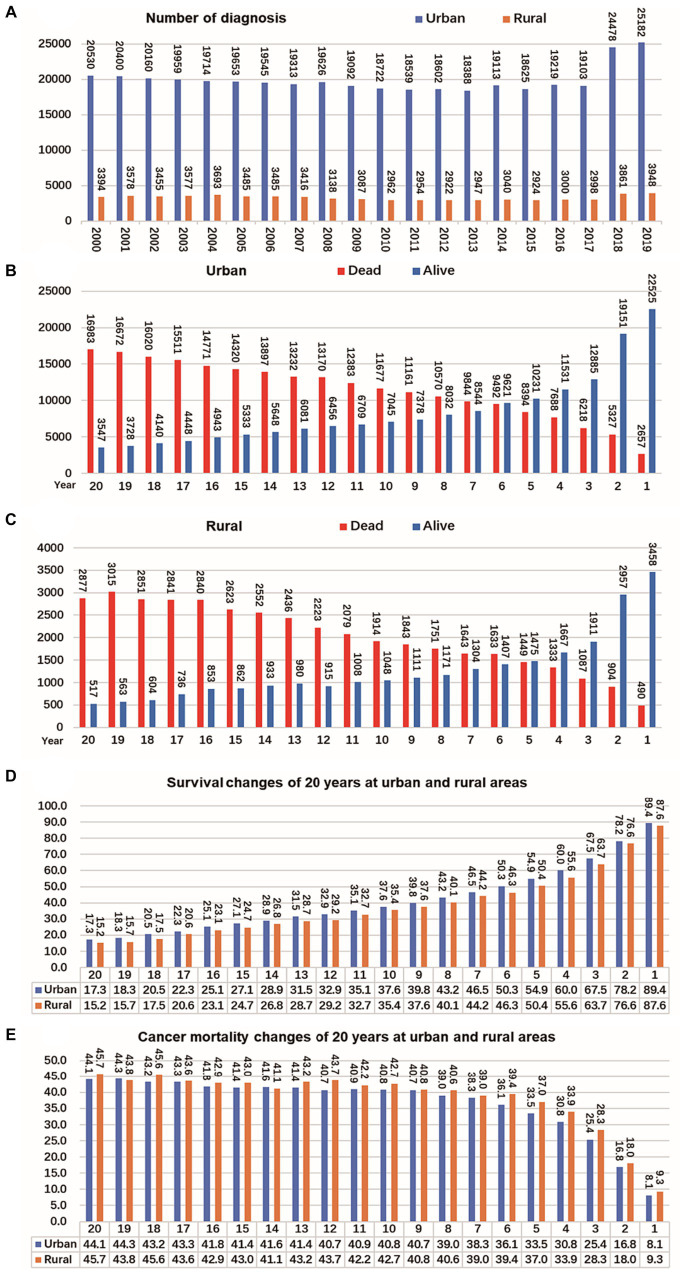
Changes of CRC cases, survival and mortality in urban and rural areas in 20 years. **(A)** Number of CRC per year from 2000 to 2019, shows that the number of cancer diagnoses in rural and urban areas remained relatively stable from 2000 to 2017, and the number of cancer diagnoses increased significantly from 2018 to 2019. **(B)** The tumor mortality rate (49.7%) and survival rate (50.3%) reached a balance in the sixth year in urban. **(C)** The tumor mortality rate (49.6%) and survival rate (50.4%) reached a balance in the fifth year in rural. **(D)** Survival of CRC changes in 20 years at urban and rural areas. **(E)** Mortality of CRC changes in 20 years at urban and rural areas.

The registration of CRC cases remained stable in both rural and urban areas between 2000 and 2017 but significantly increased from 2018 to 2019. In urban areas, the mortality and survival rates for CRC reached a balance in the sixth year after diagnosis, meaning that during the first 6 years after diagnosis, survival was higher than mortality, but after that, mortality exceeded survival. On the other hand, in rural areas, the mortality and survival rates reached a balance in the fifth year after diagnosis. Looking at the overall survival and mortality rates, CRC patients in urban areas had higher survival rates compared to those living in rural areas, while those living in rural areas had higher mortality rates than those in urban areas. These findings are shown in Figure 5.

### Trends changes of OS of CRC in urban and rural areas in 20 years

**Figure 6:**
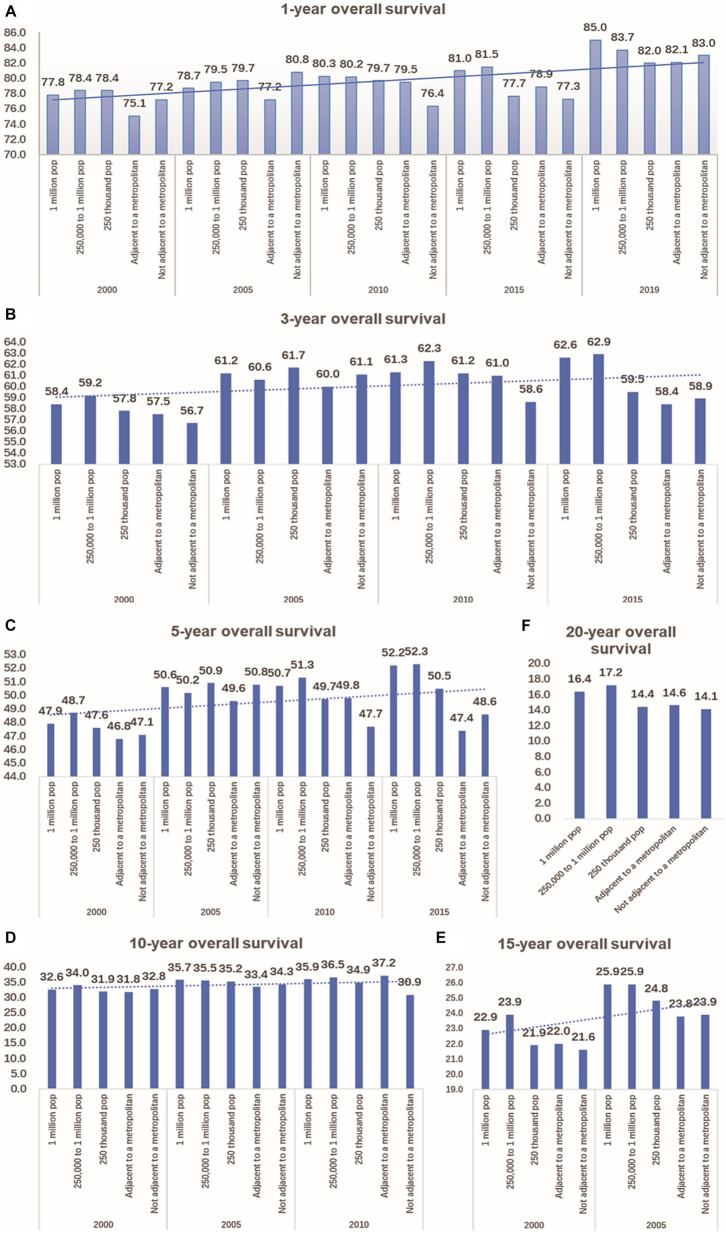
Comparison of 1-year, 3-year, 5-year, 10-year, 15-year, and 20-year OS of CRC between urban and rural in 20 years. **(A)** The change of CRC’s 1-year OS in metropolitan, medium city, small city, adjacent to a metropolitan and countryside, Compared with 20 years ago, the 1-year OS of CRC in urban and rural has significantly improved. **(B)** Compared with 15 years ago, the 3-year OS of CRC in urban and rural increased by 0.9–3.2 percentage points. **(C)** Compared with 15 years ago, the 5-year OS of CRC in urban and rural increased by 0.6–4.3 percentage points. **(D)** Compared with 2000, the 10-year OS of CRC in urban increased by 2.3–3.0 percentage points in 2010,in the adjacent to a metropolitan area, it increased by 5.4 percentage points, in the not adjacent to a metropolis area, it decreased by 1.9 percentage points. **(E)** Compared with 2000, the 15-year OS of CRC in urban and rural increased by 1.8–3.0 percentage points in 2005. **(F)** Comparison of 20-year OS of CRC in urban and rural.

According to the data, the 1-year overall survival rate of CRC has improved significantly over the past 20 years, both in urban and rural areas. In 2019, the 1-year overall survival rate of CRC in a population of 1 million was 7.2 percentage points higher than that of 2000. In Not adjacent to a metropolis, this improvement was 5.8 percentage points higher. Additionally, compared to 15 years ago, there has been an increase in the 3-year and 5-year overall survival rates of CRC by 0.9–3.2 percentage points and 0.6–4.3 percentage points, respectively, in both urban and rural areas. In terms of longer-term outcomes, there was an improvement in the 10-year overall survival rate of CRC in urban areas by 2.3–3.0 percentage points in 2010, compared to 2000. In metropolitan areas, there was a larger increase of 5.4 percentage points. However, there was a decrease of 1.9 percentage points in non-metropolitan areas (as shown in Figure 6).

## Discussion

The research findings indicate that there were notable differences in the characteristics and outcomes of CRC cases between urban and rural areas in the period between 2000 and 2019. Specifically, a higher proportion of CRC cases in the urban setting were female, black, diagnosed at advanced stages (stage III and stage IV), and had tumors less than 5 cm. Furthermore, a larger percentage of urban CRC cases had a higher household income of over $55,000, compared to their rural counterparts. In terms of mortality rates, both total and CRC-specific mortality rates were higher in rural compared to urban areas, with a 3.5 percentage point difference for total mortality and a 1.9 percentage point difference for CRC-specific mortality. It is worth noting that men had a significantly higher risk of developing and dying from CRC compared to women in the US (5). Men diagnosed with CRC have a 62.8% chance of surviving 5 years from the date of diagnosis compared with women’s 64.7% chance of survival (6). Moreover, while CRC incidence and mortality rates have decreased in both genders in China, men remained at a higher risk throughout. Factors such as smoking, obesity, alcohol consumption, and lack of physical activity contributed more to the development of CRC in men than women (7). Commonly, Chinese men smoke more frequently than women (8).

Several studies have examined the survival rates of CRC patients. One study conducted by Hashibe et al. found that rural CRC patients had lower survival rates compared to other areas (9). Additionally, statistics indicate that historically, Black men have had higher incidence and mortality rates for CRC compared to other racial and ethnic groups. The 5-year survival rate for Black individuals with CRC is reported to be 59%, while for White individuals, it is 63.8% (6). It is well-established that CRC screening can effectively prevent or detect CRC at an early stage (10). However, there are certain barriers that can hinder access to appropriate primary care services for racially minoritized populations. These barriers include lack of insurance or social support, as well as racism and discrimination (11)^.^ Studies conducted within the Veterans’ Health Administration have shown that there are no differences in diagnostic follow-up testing between White and Black individuals, suggesting that access to appropriate structures and services may be crucial in ensuring appropriate post-screening follow-up for minoritized populations (12). while considering unique social and healthcare contexts, there are cultural and community-specific approaches that can be employed to promote CRC screening and follow-up care among racially minoritized populations. Here are some our suggestions: Provide culturally tailored education: Develop educational materials and campaigns that are sensitive to the cultural beliefs, values, and practices of racially minoritized populations. Use culturally appropriate language, images, and storytelling methods to communicate the importance of CRC screening and follow-up care. Enhance community engagement: Engage community leaders, organizations, and influencers to raise awareness about CRC screening. Utilize trusted community members who can act as ambassadors and share personal stories or testimonials of their experiences with CRC screening. Provide multilingual services and assistance to avoid language barriers that prevent screening and follow-up care. Respect the Faith-based initiatives of ethnic minorities, strengthen cooperation with healthcare providers, enhance their cultural abilities and awareness of the unique needs of ethnic minorities. Establish mutual trust and provide personalized care.

The findings from a Kaplan–Meier survival analysis revealed that in the CRC group with a household income less than $35,000, rural areas had a median survival time that was 9 months longer compared to urban areas. This difference may be attributed to the higher proportion of White individuals (88.2%) in rural areas compared to urban areas (76.6%). On the other hand, when considering variables such as age, sex, race, primary site, stage 0-stage IV, tumor size, and household income over $35,000, the median survival time of CRC in urban areas was higher than in rural areas. This observation suggests that urban areas may have an advantage in terms of CRC early detection screening. It is well-known that CRC early-detection screening plays a vital role in improving survival rates (13). However, socioeconomic factors can act as barriers that hinder both the planning and completion of CRC screening (14).

Our findings indicate that the overall survival rate of CRC patients was higher in urban areas compared to rural areas. Conversely, the mortality rate of CRC was higher in rural areas compared to urban areas. In urban areas, the survival rate of CRC patients was lower than the mortality rate at the sixth year after diagnosis, while in rural areas, this occurred at the fifth year. This study suggests that when evaluating the effectiveness of CRC treatment, it may be more appropriate to assess the 6-year survival rate in urban areas and the 5-year survival rate in rural areas. Furthermore, when comparing the data from 2000, we observed a significant improvement in the 1-year, 3-year, and 5-year overall survival rates of CRC in both urban and rural areas.

Our study identified several factors that independently influenced the prognosis for the OS of CRC. These factors included age over 40 years, male gender, Black ethnicity, tumor location in the right colon, advanced stages (stage III and stage IV), and tumor size over 5 cm. Additionally, household income below $75,000 and $55,000 were found to be independent prognostic factors for the OS and DSS of CRC in urban and rural areas, respectively. Overall, this study highlights various risk factors that impact the survival of CRC patients, including demographic characteristics like age, gender, and race, as well as medical factors such as tumor location, stage, and size. The study also emphasizes the importance of socioeconomic status, as household income was found to significantly impact CRC survival. There is a growing concern worldwide about the increasing incidence of CRC in younger adults (below 50 years old). This trend has raised clinical concerns that younger adults may present with more advanced disease, leading to a poorer prognosis compared to older cohorts due to a lack of screening (15, 16). Recent studies have reported that a younger age at diagnosis and receiving systematic therapies could potentially result in longer OS and DSS for CRC patients (17). The distribution of CRC varies significantly across different regions worldwide (18). It is predominantly observed in Australia, Europe, and North America. In general, the incidence in developed countries or regions is approximately three times higher than in less developed areas. However, there is a notable increase in the incidence of CRC in Asia that cannot be ignored (19, 20). In China, both the incidence and mortality rates of CRC have shown an upward trend over the years. According to data from the Chinese Cancer Registration in 2014, the highest incidence and mortality rates were observed in the eastern region, followed by the central region, with the lowest rates in the western region. The mortality rate of colorectal cancer in urban areas of China experienced a significant increase from 2002 to 2008, followed by a decrease from 2008 to 2015. Conversely, the mortality rate in rural areas continued to rise (21). The specific reasons for this change are not very clear, and we think it may be related to the imbalance in economic development between urban and rural areas in China. Researchers believe that it is related to the following factors, such as inequitable distribution of health care services between urban and rural areas; pilot CRC screening strategies were put into place by the Chinese government in 2012. However, these programs were conducted only in urban areas; rural areas generally lack adequate field conditions, implementation funding, and screening equipment (21). We can be learned from the lessons for future public health strategies as follow, Providing accessible healthcare services: Ensuring access to high-quality healthcare facilities and services, especially in rural areas, can promote early diagnosis and effective treatment of CRC. Develop corresponding screening plans for urban and rural areas to ensure that high-risk populations in both areas receive appropriate screening. It is recommended to conduct regular screening for individuals with moderate risk, while individuals with higher risk may require earlier or more frequent screening. Promoting a healthy lifestyle: Encouraging individuals to develop healthy habits, such as regular physical activity, maintaining a balanced diet rich in fruits and vegetables, limiting the consumption of processed foods, avoiding smoking and excessive alcohol consumption, can reduce the risk of developing CRC.

## Limitations and strengths

This study has certain limitations and strengths that should be considered. One limitation is that the data used in this study is derived from the SEER database, which represents the US population. Therefore, the generalizability of the findings to other countries or regions may be limited. Additionally, the lack of treatment data in the SEER database restricts the ability to compare the impact of different treatments on prognosis in urban and rural areas. Despite these limitations, the study has notable strengths. One strength is the inclusion of 20 years of urban and rural data, allowing for an analysis of the changes in survival and prognostic factors for CRC over this period. Moreover, the study highlights the importance of considering different survival rates for urban (6-year survival) and rural (5-year survival) populations when evaluating treatment effects. Further research is needed to validate these findings in diverse settings and to explore the impact of specific treatments on the prognosis of CRC in urban and rural areas.

## Conclusion

To summarize, our study revealed that the OS of urban CRC patients is slightly lower than that of rural patients, while, there was no significant difference in DSS between urban and rural CRC patients. In urban areas, the mortality rate of CRC exceeded the survival rate in the sixth year after diagnosis, while in rural areas, it was the fifth year after diagnosis. Over the past 20 years, there has been an improvement in the 5-year OS of CRC, with an increase of 2.9–4.3 percentage points in urban areas and 0.6–1.5 percentage points in rural areas. Several independent prognostic factors for OS of CRC were identified in both urban and rural settings. These factors included age over 40 years, male gender, Black ethnicity, and tumor size over 5 cm. Additionally, household income below $75,000 and below $55,000 were found to be independent prognostic factors for OS and DSS of CRC in urban and rural areas, respectively.

These findings highlight the importance of considering urban–rural disparities in CRC prognosis and the influence of socioeconomic factors on survival outcomes. Further research is needed to explore the underlying reasons for these disparities and to develop targeted interventions to improve outcomes for CRC patients in both urban and rural settings.

## Data availability statement

The original contributions presented in the study are included in the article/supplementary material, further inquiries can be directed to the corresponding author.

## Ethics statement

Ethical approval was not required for the study involving humans in accordance with the local legislation and institutional requirements. Written informed consent to participate in this study was not required from the participants or the participants’ legal guardians/next of kin in accordance with the national legislation and the institutional requirements.

## Author contributions

M-sF: Conceptualization, Data curation, Formal analysis, Investigation, Methodology, Project administration, Visualization, Writing – original draft, Writing – review & editing. S-xP: Data curation, Project administration, Visualization, Writing – review & editing. X-qC: Formal analysis, Visualization, Data curation, Writing – review & editing. Q-cP: Resources, Supervision, Writing – review & editing.
